# Supplementary material for: Development and optimization of red spectrum splitting concentrated agrivoltaic system for energy generation and sustainable agriculture
Source: PLoS One. 2025 Sep 22;20(9):e0332865. doi: 10.1371/journal.pone.0332865 (PMC12453254; doi:10.1371/journal.pone.0332865)
Supplement: Attachment — Submitted filename: PONE-D-25-24618.docx [file pone.0332865.s001.docx]

**Journal Name: PLOS ONE**

**Title:** Development and Optimization of Red Spectrum Splitting Concentrated Agrivoltaic

System for Energy Generation and Sustainable Agriculture

Manuscript ID: PONE-D-25-24618

This This paper introduces a Red Spectrum Splitting Concentrated Agrivoltaic (RSSCA) system that enhances both solar power generation and rice cultivation by directing red light to crops and other wavelengths to solar cells. The system achieves 31.2% efficiency and supports healthy rice growth, making it more effective than conventional PV systems. However, in its current state, the manuscript is not suitable for publication in the PLOS ONE journal. Need to address the comments below mentioned:

The comments are:

[1] Abstract should cover the problem statement, objectives, methodology, discussion, important outcome and significance of the review.
[2] Add minimum 4-5 keywords
[3] There is no flow in the introduction part. In the introduction, clearly address the problem and related studies can be discussed. Very less literature has been discussed. Improve the literature part. Also, The sources are not properly cited, and there are no supporting references provided for the first two paragraphs.
[4] Novelty part should be revised (last paragraph of introduction). At the end of the introduction, provide a strong discussion on the significance of this study. Highlight the existing research in this field and clearly identify the specific gap that your research aims to address.
[5] The structure of the article different from journal guidelines, follow the journal guidelines, (like Introduction, Materials and methodology, results and discussion and Conclusion section)
[6] The equations not derived by should be cited the proper source.
[7] The manuscript should include a comparative analysis of PV power generation and paddy crop yield outcomes to validate the effectiveness of the proposed system.
[8] The Results and Discussion section lacks clarity, as it is currently mixed with methodology details. Please separate and clearly distinguish the methodology from the results and their interpretation to improve readability and coherence
[9] The explanations of figures 11, 12 and 13 not enough, you have to discuss strongly with scientific reason
[10] Expand the literature review to include more recent studies and clearly identify the gap your research is addressing. There is no studies from 2022-2024 and no many recent studies.
[11] Conclusion should be improved by incorporating future recommendations, addressing limitations, and emphasizing the significance of the study.
[12] The level of English language should be checked throughout the manuscript and to make sure that the article is free from grammatical mistakes.
[13] Recommended to add 4-5 highlights. Highlights should be 85 characters no spaces (follow the journal guidelines).
[14] Draw a graphical abstract related to your research. It should be simple and meaningful, It helps the readers identify more quickly which papers are most relevant to their research interest(if Possible).
